# Agent Orange: Haft-Century Effects On The Vietnamese Wildlife Have Been Ignored

**DOI:** 10.1021/acs.est.1c06613

**Published:** 2021-10-29

**Authors:** Kiem N. Truong, Khuong V. Dinh

**Affiliations:** † Department of Ecology, Faculty of Biology, University of Science, Vietnam National University, VNU Hanoi, 334 Nguyen Trai, Thanh Xuan, Ha Noi 10000, Vietnam; ‡ Section for Aquatic Biology and Toxicology, Department of Biosciences, University of Oslo, Blindern, PO Box 1066, 0316 Oslo, Norway

**Keywords:** Agent Orange, wildlife, dioxin

The application of more than 91 million liters of Agent Orange in Vietnam defoliated ∼3.1 million hectares of biologically diverse tropical forests and mangroves from Quang Tri to Ca Mau (>1000 km) in 1961–1971. The last application of Agent Orange in Vietnam was over for at least five decades, yet more than 4 million people, particularly local people and veterans have suffered and died from various types of cancers and congenital disabilities^1^ (see also the references in Supporting Information (SI) S1). A persistent and long-lasting effect component of Agent Orange is 2,3,7,8-tetrachlorodibenzo-p-dioxin (2,3,7,8-tetraCDD), the most toxic congener of dioxin. Dioxin and dioxin-like substances are still on the list of 10 chemicals of concern of the World Health Organization of the United Nations.^2^ So far, the dioxin effects on exposed people have lasted for at least three generations.^3^ The levels of dioxin in the breast milk of women^4^ and serum of men^5^ in sprayed regions are still several times higher than in those in nonsprayed areas, suggesting the potential effects for the next generation(s) to come.

Although Agent Orange was sprayed across large areas of biodiversity hotspots and priority regions for conservation,^6^ its effects on Vietnamese wildlife have been ignored in most, if not all, previous investigations. Indeed, a number of studies have documented Agent Orange effects on human health and healthcare-related issues, fewer studies on its accumulation in foods, water, sediments, and soils, and some poor documentations on the recovery of vegetation (Figure 1 and references in SI S1). Strikingly, none has tapped into the effects of Agent Orange substances on the biodiversity changes and evolutionary responses of sprayed fauna (Figure 1). This is alarming as high levels of dioxin in the soil, water, and sediments^7^ could seriously affect Vietnamese wildlife animals.

**Figure 1:**
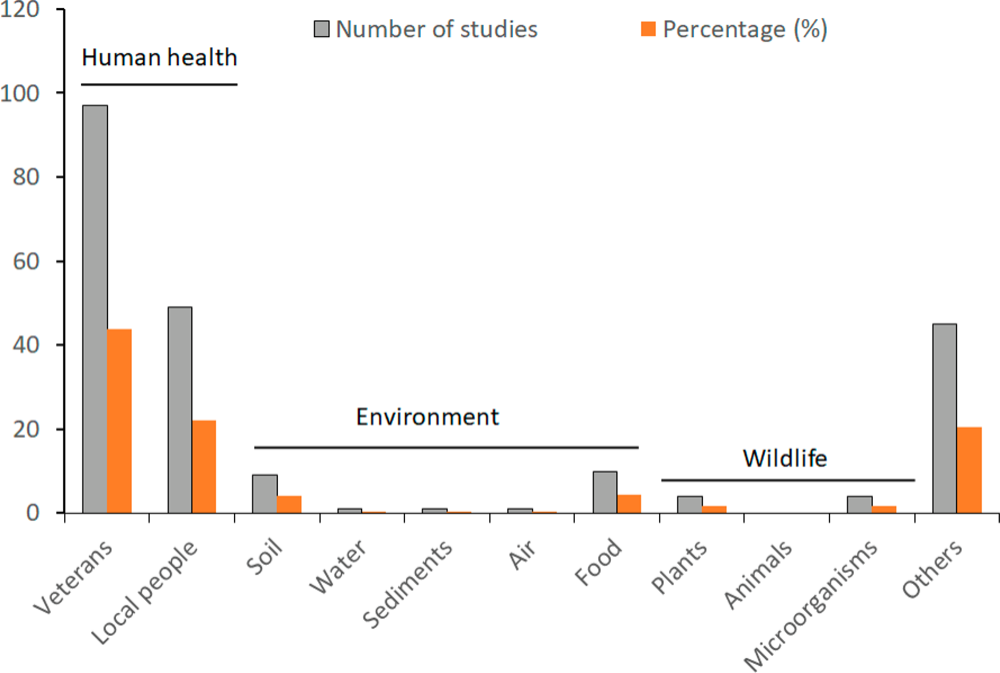
Overview of studies on the effects of Agent Orange on humans, environment, wildlife, and others in the last 50 years. Other studies were mainly diplomatic, justice, social and military sciences, and health care.

Recently, dioxin cleanup programs have been initiated and partly completed in several small “hotspots” of Agent Orange, such as Da Nang Airport and Bien Hoa Airbase areas.^7^ This is an important step to reduce and mitigate the risk of Agent Orange exposure and its effects on local people. However, together with the cleanup programs, there is an urgent need for comprehensive investigations of Agent Orange effects on local wildlife before entire aquatic, benthic, soil animals, fungi, and bacteria in sprayed regions, particularly in hotspots may be destroyed, for example, in heating contaminated soils and sediments to high temperature (∼335 °C). Furthermore, the vast majority of >1000 km sprayed regions scattering in central and southern Vietnam remains to be investigated. Five key issues need to be considered, including

(1) How Agent Orange has changed the diversity of sprayed fauna, thereby altering structure and function of sprayed ecosystem? Aquatic, benthic, and soil invertebrates generally have low mobility and could not escape from exposure to Agent Orange substances. Key shredders and grazers, but also pollution sensitive taxa such as Ephemeroptera, Plecoptera, Trichoptera (EPT), and Cladocera, may be highly vulnerable to Agent Orange substances, thereby substantial changes in their species composition, population structure, and dynamics, and the cascading effects on the entire sprayed ecosystems would be expected. Biodiversity changes can be revealed by comparative studies on fauna biodiversity and physiology in sprayed and nonsprayed locations across regions with different levels of Agent Orange applications.
(2) The impact of Agent Orange on soil fauna: Soil animals such as earthworms, centipedes, millipedes, springtails, termites, and ants are key components of geo-biochemical cycles and carbon sequestration in the soil, especially in tropical rain forests. Agent Orange effects on ecological biodiversity and functions of these soil animals may alter the soil fertility, carbon sequestration, local and regional climate, but these critically novel and important issues remain to be investigated and addressed.
(3) The impact of Agent Orange on higher wildlife animals: It is unknown whether there have been congenital malformations of the higher wildlife animals such as birds and mammals in sprayed regions. This concern stems from observations of various congenital malformations of exposed people.^1^ Notably, many Vietnamese birds (e.g., Vietnam pheasant - *Lophura edwardsi*), and mammals (e.g., Sao La - *Pseudoryx nghetinhensis*) are endemic and have limited distributions within or overlapped with the sprayed regions. These Vietnamese wildlife animals already face a high risk of extinction by various anthropogenic activities such as land use, deforestation, and element extractions.
(4) Potential evolutionary responses of exposed animals to Agent Orange: mechanisms, costs and ecological consequences: Surviving animals must develop an increased tolerance to Agent Orange substances. Evolution of increased tolerance is likely after 50–60 years of exposure to Agent Orange substances, equivalents to several hundred to thousand generations of short-lived aquatic and soil invertebrates, for excample, cladocerans.^8^ Adaptations of Vietnamese invertebrates to long-term exposure to metals have recently been observed.^9^ Evolutionary responses of surviving animals to Agent Orange can provide unique insights into physiological, epigenetic and genetic mechanisms underpinning the long-term effects of Agent Orange on the hyperdiverse tropical ecosystems such as sprayed forests, lakes, and rivers. Specimens can be sampled from various locations between sprayed (Ma Da Area) and nonspray regions (e.g., Cat Tien National Park) and analyzed to explore potential genetic changes. Common garden experiments using populations collected along Agent Orange gradients can also be applied to examine the cost of adaptations and the capacity of these species to deal with other major drivers of biodiversity loss such as climate change.
(5) Changes of ecosystem resiliencies to emerging local and global stressors: Long-term exposure to Agent Orange may result in changes in susceptability of local, particularly endemic species to other major stressors for biodiversity loss such as land use, fragmented habitats, agriculture, industrial, and urbanization development and climate change; which may collapse entire hyperdiverse tropical ecosystems.^10^ In fact, Vietnam has shown substantial biodiversity loss in the last decades^11,12^ while it is also one of most vulnerable countries to climate change.^13^ Studying the impacts of Agent Orange on wildlife is highly valuable and will have far-reaching applications for ecotoxicological risk assessments of Agent Orange and other persistent organic pollutants (POPs). How long-lasting chemicals work in concert with climate change to affect Vietnamese wildlife is largely unknown.^8,14^

We here raise an untapped but critically important issue about the effect of Agent Orange, one of the most devastating and long-lasting environmental issues in human history. It is critically important to have support from both the Vietnamese and U.S. Governments to include environmental assessments and biodiversity protection as part of the cleanup programs.^7^ Participation of United Nations Environment Programs (UNEP), International Union for Conservation of Nature (IUCN), World Wildlife for Funds (WWF), the scientific community, and local people are essential to provide a diversity of required expertise for environmental risk assessments, environmental justice, and biodiversity protection of exposed wildlife.

## Supporting Information Available

A list of published papers on Agent Orange. Data were collected from the Web of Science (August 2021) (XLSX)
